# Spy1/SpeedyA accelerates neuroblastoma

**DOI:** 10.18632/oncotarget.2380

**Published:** 2014-08-21

**Authors:** Xavier Bisteau, Philipp Kaldis

**Affiliations:** Institute of Molecular and Cell Biology (IMCB), A*STAR (Agency for Science, Technology and Research), Proteos#3-09, and National University of Singapore (NUS), Department of Biochemistry, Singapore, Republic of Singapore

Contrary to malignant brain tumors arising in adults such as glioma, neuroblastoma develops in young children and is the most common aggressive cancer diagnosed in the first year after birth. Neuroblastoma is an embryonic tumor originating from the migratory neural crest and arises in tissues of the sympathetic nervous system (Maris, N. Engl. J. Med. 2010; 362, 2202). Because of its origin, neuroblastoma exhibits a large variety of cell types classified in three main populations (N, S and I). Among them, I type cells are neuroblastoma stem cells with differentiation and malignant potential and are considered “tumor initiating cells” (TIC). They typically express stem cell markers such as CD133 and c-kit, are capable of self renewal, and form fast growing tumors (Ross and Spengler, Semin. Cancer Biol. 2007; *17*, 241). In this context, the balance between proliferation and differentiation of this cell population remains unclear but is tightly linked to the cell cycle progression. Whereas numerous cell cycle regulators are involved in driving proliferation, the recently characterized cyclin-like protein, Spy1 (SpeedyA), is tightly regulated during proliferation of different progenitor cells such as mammary cells or astrocytes (Golipour A et al., Cancer Res. 2008; 68: 3591). Furthermore, Spy1/SpeedyA levels were elevated in malignant human glioma correlating with a poor survival prognosis.

To decipher the implication of Spy1/SpeedyA in proliferation of neuroblastoma and its potential role in the TIC, Lubanska et al. analyzed several neuroblastoma cell lines (Lubanska and Porter, Oncoscience. 2014; *1*: 336). The expression of Spy1/SpeedyA significantly differs between the tested cell lines, with CHLA-15 and CHLA-20 cell lines expressing higher Spy1/SpeedyA protein levels in comparison to SH-SY5Y cells. In this context, SH-SY5Y treated with retinoic acid to induce neural differentiation displayed a gradual decrease of Spy1/ SpeedyA expression as cells differentiated. This decrease in expression of Spy1/SpeedyA was accompanied by the downregulation of the neural stem cell marker Nestin. Conversely, the overexpression of Spy1/SpeedyA by transient transfection in SH-SY5Y cells resulted in a delay of the induced differentiation, enhancing proliferation as observed by an increased number of cells and increased BrdU and PCNA staining.

The lack of differentiation was correlated with increased proliferation of Spy1/SpeedyA overexpressing cells, leading the authors to evaluate the self-renewal capacity of SH-SY5Y cells by their ability to form neurospheres. Overexpression of Spy1/SpeedyA in SH-SY5Y cells increased the number of spheres smaller than 100μm. Moreover, increased expression of Spy1/SpeedyA leads to increased expression of markers for pluripotency (Oct-4), stem cells (Bmi1), glial progenitors (OLIG2), and astrocytes (GFAP). Interestingly, Spy1/SpeedyA also enhanced the expression of the TIC marker, CD133, whereas its knockdown in CHLA-15 and CHLA-20 cells reduced CD133 expression, the number of spheres, and their proliferation. To validate the hypothesis of a direct relation between CD133 and Spy1/SpeedyA expression, the authors sorted CD133^+^ and CD133^−^ populations of CHLA-15 cells and modulated Spy1/SpeedyA expression. There was an increase of spheres associated with a higher level of CD133 after overexpression of Spy1/SpeedyA in CD133^−^ population, and the knockdown of Spy1/SpeedyA in CD133^+^ population reduced drastically the number of spheres and their proliferation.

The relation between Spy1/SpeedyA, CD133 expression and TIC described by Lubanska et al. in neuroblastoma cells is similar to their previous observations in glioma cell lines (Lubanska et al., Cancer Cell. 2014; *25*: 64). Despite differences in the expression of specific markers between both tumor types, elevated Spy1/SpeedyA expression correlated in both cases with a higher proliferation of the CD133^+^ population. By knocking down Spy1/SpeedyA in multiple glioma cell lines, they observed an increase in asymmetric divisions correlated with the localization of Numb. This could explain the increased differentiation observed under the condition of reduced level of Spy1/SpeedyA. Furthermore, determination of the Spy1/SpeedyA protein levels in multiple types of glioma revealed an increased expression in approximately 60% of tested tumors, which correlates with the increasing tumor grade as well as a reduction of patient survival. Collectively, these observations lead to the conclusion that Spy1/SpeedyA is an important factor in proliferation, especially in progenitor cells and tumor initiating cells.

Spy1/SpeedyA belongs to a family of “cyclin-like” proteins that are differentially expressed during development and in a tissue specific manner (Chauhan et al., Cell. Mol. Life Sci. 2012; *69*: 3835). Increased expression of Spy1/SpeedyA has been observed in highly proliferative cells during development as well as in cancer. To this end, Spy1/SpeedyA interacts and activates Cdk2 allowing Cdk2 to phosphorylate a large range of substrates and bypassing some of the classical cell cycle checkpoints (Cheng et al., BMC Biochem. 2005; *6*, 19). It is not clear whether Spy1/SpeedyA acts in a Cdk2 dependent or independent fashion in neuroblastoma. Therefore, the identification of Spy1/SpeedyA as a positive driver of CD133^+^ population could lead to a new therapeutic approach against highly aggressive and multidrug resistant neuroblastoma and glioma. However, the mechanism how the Spy1/SpeedyA protein increases the expression of TIC markers, self-renewal, and proliferative rate in comparison to other cyclins binding to Cdk2 will need elucidated in the future.

